# A Less Invasive Technique for Surfactant Administration in a Full-Term Newborn With Moderate Meconium Aspiration Syndrome

**DOI:** 10.7759/cureus.57293

**Published:** 2024-03-30

**Authors:** Nuor Abusallout, Sarah Abdulrahman, Ahmed Elhadidi, Aimen Ben Ayad

**Affiliations:** 1 Neonatology, Tawam Hospital, Al Ain, ARE

**Keywords:** surfactant, respiratory distress, neonate, meconium, mas, lisa

## Abstract

Meconium aspiration syndrome (MAS) presents significant challenges in neonatal care, particularly in the context of respiratory distress. This report explores the efficacy of administering surfactant through a less invasive surfactant administration (LISA) technique in a full-term neonate with MAS. Our case highlights the potential of this method in reducing the need for mechanical ventilation, drawing parallels with its established benefits in preterm neonates with respiratory distress syndrome. The successful application of LISA in this case suggests a promising avenue for managing MAS in full-term neonates, warranting further investigation.

## Introduction

Meconium aspiration syndrome (MAS) is a critical neonatal condition characterized by respiratory distress in newborns exposed to meconium-stained amniotic fluid, with distinct radiological features [1]. Accounting for approximately 10% of neonatal respiratory failures [2], MAS incidence escalates from 38 to 42 weeks of gestation [3]. Although its prevalence has declined in developed countries, MAS remains a significant challenge in developing regions due to disparities in obstetric and perinatal care.

The management of MAS includes a multidisciplinary approach, aiming at stabilizing the neonate hemodynamically, optimizing lung function, and minimizing the risk of persistent pulmonary hypertension and iatrogenic injuries. A notable advancement in this domain is the utilization of surfactant therapy. Surfactant plays a crucial role in ameliorating MAS severity and reducing the necessity for extracorporeal membrane oxygenation (ECMO), as evidenced by Cochrane reviews [4]. The American Academy of Pediatrics Committee on Fetus and Newborn advocates surfactant use in MAS for improving oxygenation and diminishing ECMO requirements [5].

Traditionally, surfactant delivery is achieved via endotracheal tubes; however, less invasive methods like LISA (less invasive surfactant administration) or MIST (minimally invasive surfactant therapy) are gaining acceptance. These techniques, involving the administration of surfactant through a thin catheter, have demonstrated promising outcomes in preterm neonates with respiratory distress syndrome (RDS), showing a reduction in mortality and bronchopulmonary dysplasia (BPD) risk [6]. Nevertheless, the literature on the application of LISA/MIST in full-term neonates with MAS remains sparse.

This report presents a case of a full-term infant with MAS, where the application of surfactant via the LISA technique circumvented the need for mechanical ventilation and mitigated subsequent MAS complications.

## Case presentation

The patient, a female newborn, was born to a 32-year-old primigravida mother. The expected delivery date was July 15, 2022. The mother had limited prenatal care and was not previously registered at our facility. Her serological tests, including Group B Streptococcus (GBS), were negative.

The mother presented to the emergency department of Tawam Hospital on July 21, 2022, one week post-term, with symptoms of abdominal pain, decreased fetal movement, and greenish vaginal discharge. Initial examination in the emergency room revealed hypertension (140/89 mmHg), lower limb edema, and brisk deep tendon reflexes, but no headache or blurred vision was reported. A vaginal examination indicated a dilated cervix at 3 cm, with no amniotic membrane present. Cardiotocography (CTG) showed a fetal heart rate of 140 bpm with two late decelerations and reduced variability (<5 bpm).

A diagnosis of active labor with meconium-stained amniotic fluid and pre-eclampsia was made, alongside non-reassuring fetal heart rate patterns. Blood pressure management was initiated with hydralazine. Given the rapid progression of labor, an emergency lower segment cesarean section under spinal anesthesia was performed due to cord presentation and non-reassuring CTG.

At birth, the newborn appeared pale and hypotonic, with poor respiratory efforts, and was covered with meconium staining. Immediate resuscitative measures were undertaken, including suctioning of the mouth and nose, revealing thick meconium. The heart rate was above 60 bpm, with positive pressure ventilation, and then the heart rate improved to over 100 bpm. Continuous positive airway pressure (CPAP) was initiated at three minutes of age as the infant's respiratory efforts and tone began to improve. Apgar scores were 4 at 1 minute, 7 at 5 minutes, and 9 at 10 minutes.

The newborn was subsequently transferred to the Neonatal Intensive Care Unit (NICU) on CPAP with 30% FiO_2_, presenting with moderate respiratory distress. Clinical parameters included a respiratory rate of 55 - 57/min, a heart rate of 165/min, and oxygen saturation of 88 - 94%. Non-invasive ventilation was initiated (NIV PC: PIP of 22 cmH_2_O, PEEP of 5 cmH_2_O, FiO_2_ 35%). Empirical antibiotic therapy with penicillin G and gentamicin was started, pending blood culture results, to address potential early-onset sepsis.

**Figure 1:**
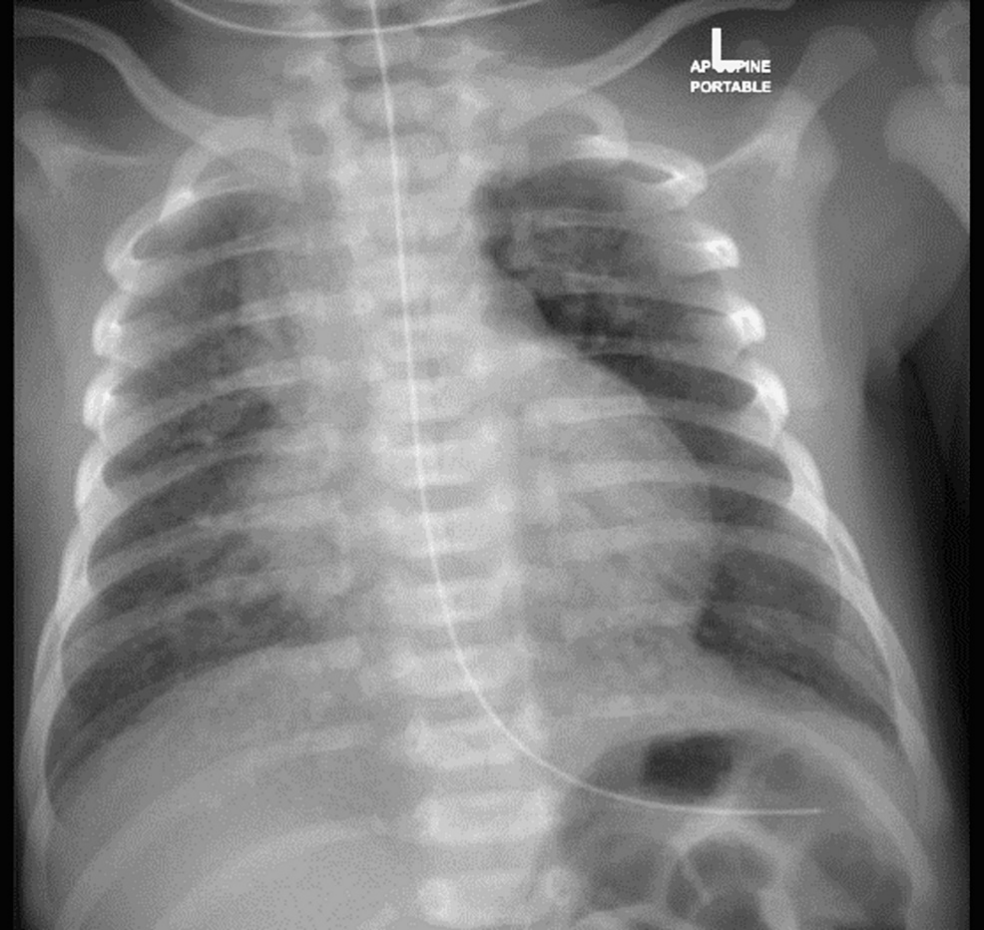
First chest X-ray before LISA LISA: Less invasive surfactant administration

**Figure 2:**
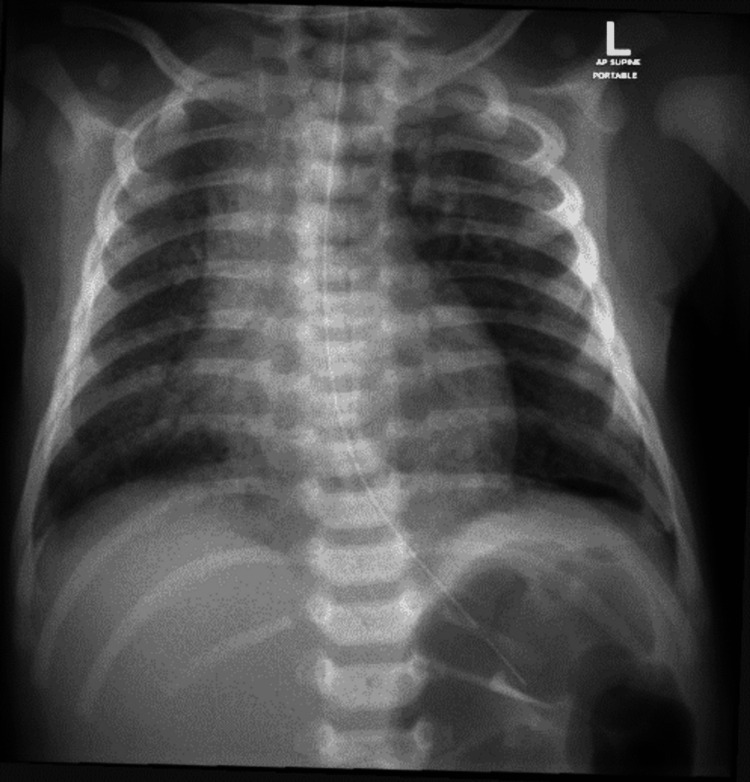
Chest X-ray 14 hours post LISA LISA: Less invasive surfactant administration

**Figure 3:**
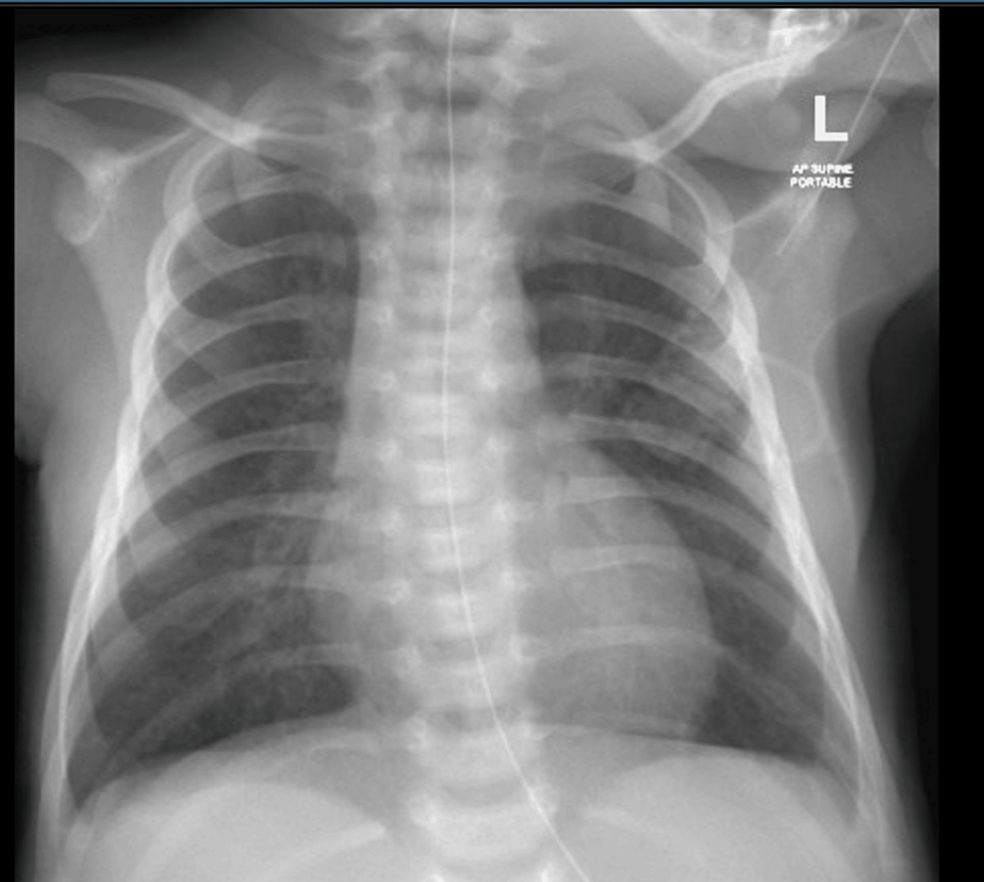
Chest X-ray before discharge

Cord blood gas venous: PH 7.21, PCO_2_ 47.9, HCO_3_ 19, base -9.2, lactate 7.5. A capillary blood gas analysis two hours postnatally showed metabolic acidosis (PH 7.21, PCO_2_ 38.8 mmHg, HCO_3_ 15 mmol/L, BASE -11.9 mmol/L, Hgb 191 g/L, lactate 10.8 mmol/L) and elevated lactate levels (10.8 mmol/L) (Table 1). Chest radiography revealed ill-defined hazy opacities in the paracardiac regions of both lungs and possible small pneumothorax, suggestive of MAS (Figures 1-3).

**Table 1:** Lab results

Parameter	Result	Normal Range	Units
First Blood Pressure recorded	59/37	Systolic 58-72, Diastolic 35 - 45	mmHg
Fetal Heart Rate	140	110-160	bpm
Apgar Score (1 min)	4	07-Oct	N/A
Apgar Score (5 min)	7	07-Oct	N/A
Apgar Score (10 min)	9	07-Oct	N/A
Respiratory Rate Range	55-78	30-60	breaths/min
Heart Rate	165	120-160	bpm
Preductal Oxygen Saturation	88-94	95-100	%
Blood pH	7.21	7.35-7.45	N/A
Partial Pressure of Carbon Dioxide (PCO_2_)	38.8	35 to 45	mmHg
Bicarbonate (HCO_3_)	15	22-26	mmol/L
Base Excess (BE)	-11.9	-2 to 2	mmoL/L
Lactate	10.8	0.5-2.2	mmol/L
Continuous Positive Airway Pressure (CPAP)	30% FiO2	-	%
Peak Inspiratory Pressure (PIP)	22	-	cmH_2_O
Positive End-Expiratory Pressure (PEEP)	5	-	cmH_2_O
Fraction of Inspired Oxygen (FiO_2_) for NIV	35 - 45%	21-100	%
C-Reactive Protein (CRP)	55	<10	mg/L
Hemoglobin (Hgb)	191	135 to 195	g/L

**Figure 4:**
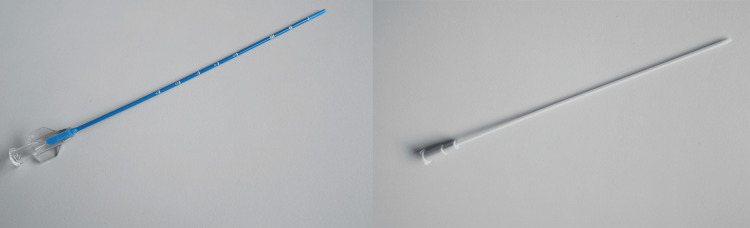
LISACath straight catheter

**Figure 5:**
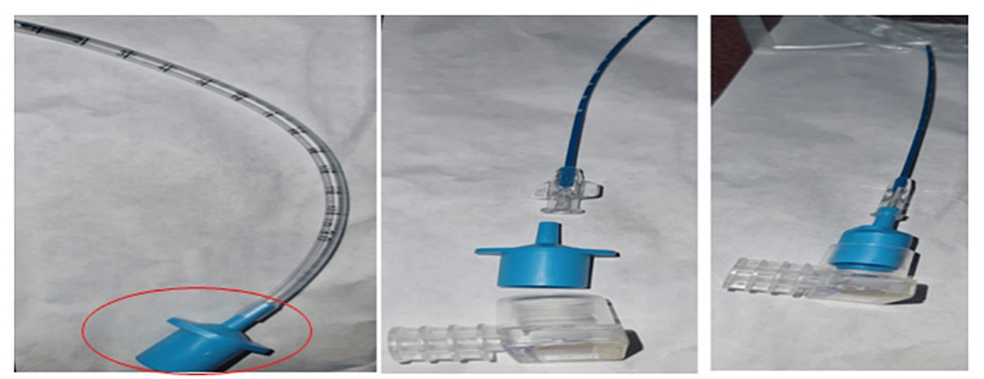
LISA catheter airway placement with CO2 detector integration

The worsening respiratory distress and increased oxygen requirements (FiO_2_ >45%) necessitated the administration of surfactant. At 15 hours of life, a single dose of Curosurf (Poractant alfa, 2.5 ml/kg) was administered via the LISA method. Post-administration, the neonate remained on NIV PC 25/7 with a gradual decrease in oxygen requirements (FiO_2_ decreased down to 35%). An echocardiogram showed normal cardiac anatomy without signs of pulmonary hypertension (Figures 4, 5).

Following LISA, the infant developed mild stridor, which was managed with racemic epinephrine administration twice. Subsequent chest radiographs showed mild diffuse bilateral opacities and a small pneumomediastinum, with improvement observed in subsequent imaging (Figure 1, 2).

The infant required non-invasive ventilation for the first 10 days of life, with gradual weaning of pressure and FiO_2_ requirements. On day 10, she was transitioned to a high-flow nasal cannula at 4 LPM with FiO_2_ 21%, which was further weaned to room air over the next three weeks. Repeat chest X-rays at two weeks showed significant improvement with no evidence of lung collapse, consolidation, opacity, or pneumomediastinum. Antibiotic treatment was continued for seven days based on clinical presentation and high CRP levels (55 mg/l), despite negative blood cultures after 48 hours, treating as clinical sepsis.

At 22 days of life, the infant was discharged home in stable condition on room air with full oral feeding.

## Discussion

This case report highlights the successful application of the LISA technique in a full-term neonate with moderate MAS. LISA, conventionally used in preterm neonates with RDS, involves administering surfactant via a thin catheter, potentially mitigating the risks associated with more invasive procedures. The avoidance of mechanical ventilation and consequent complications such as tension pneumothorax in this case highlight the potential benefit of LISA in full-term neonates with MAS.

The utilization of LISA for surfactant administration in MAS is relatively novel, particularly in full-term infants. Existing literature predominantly focuses on its use in preterm neonates with RDS, where it has been shown to improve short-term outcomes and reduce the risk of death or BPD [6]. This case contributes to the nascent body of evidence suggesting that the benefits of LISA may extend to full-term neonates with MAS, a domain where literature is currently sparse.

Several aspects of the LISA technique warrant attention. Firstly, the use of a straight catheter (LISACath™, Chiesi Farmaceutici S.p.A, Parma, Italy) facilitates a less invasive approach compared to traditional endotracheal tube delivery. Premedication with atropine, as observed in this case, has been noted to decrease the incidence of bradycardia associated with the procedure [5]. Additionally, the choice of surfactant (Poractant, Curosurf) and its dosage (200 mg/kg/dose) align with current recommendations for optimal efficacy while minimizing instillation volume.

The advantages of LISA extend beyond its less invasive nature. It allows for continued spontaneous breathing and maintenance of functional residual capacity, potentially leading to better oxygenation and ventilation dynamics. This technique also aligns with current trends in neonatal care emphasizing minimally invasive procedures to reduce iatrogenic trauma and subsequent complications.

However, this case report should be interpreted in the context of its limitations. It represents a single case, and while the outcomes are promising, broader studies are necessary to establish the efficacy and safety of LISA in full-term neonates with MAS. Controlled trials focusing on short- and long-term outcomes in this specific demographic are needed to validate the findings observed in this case.

## Conclusions

This case of a full-term neonate with MAS treated successfully with LISA highlights a potentially valuable therapeutic approach. It suggests that LISA could be a viable option in the management of MAS in full-term neonates, emphasizing the need for further research in this area. As the landscape of neonatal care evolves, techniques like LISA, which offer less invasive yet effective interventions, are likely to become increasingly important in the management of complex conditions like MAS.
